# Effect of adenoid size on the post-adenoidectomy hypernasality in children with a normal palate

**DOI:** 10.1007/s00405-023-08049-y

**Published:** 2023-06-10

**Authors:** Mosaad Abdel-Aziz, Aisha Fawzy Abdel Hady, Ayatallah Raouf Sheikhany, Ahmed Ibrahim Yousef, Omar Aly Sabry, Heba Mahomoud Farag

**Affiliations:** 1 Department of Otolaryngology, KasrAlainy Faculty of Medicine, Cairo University, 2 El-Salam St., King Faisal, Above El-Baraka Bank, Giza, Cairo, Egypt; 2 Department of Otolaryngology (Unit of Phoniatrics), KasrAlainy Faculty of Medicine, Cairo University, Cairo, Egypt

**Keywords:** Transient hypernasality, Adenoidectomy, Adenoid hypertrophy, Nasometry, Speech

## Abstract

**Purpose:**

Adenoidectomy, either alone or with tonsillectomy, is a common surgical procedure in the field of pediatric otorhinolaryngology. Resonance function may be altered postoperatively in the form of hypernasality, which is usually transient. This study aimed to investigate the effect of adenoid size on post-adenoidectomy hypernasality in children with a normal palate.

**Methods:**

Seventy-one children with different degrees of adenoid hypertrophy were included in this prospective observational study. Endoscopic assessment of the adenoid size and preoperative and postoperative evaluation of speech (at 1 and 3 months) with auditory perceptual assessment (APA) and nasometry were performed.

**Results:**

APA showed preoperative hyponasality in 59.1% of children and was found to be significantly related to the adenoid size, with more hyponasality in grades 3 and 4. One month postoperatively, hypernasality was detected in 26.7% of patients and was found to be related to the preoperative adenoid size with higher hypernasality in grades 3 and 4. Three months postoperatively, all patients had gained normal nasality except one (1.4%) who was subjected to a longer follow-up period. Nasometric assessment showed significant differences at the three visits (pre, 1, and 3 months postoperatively), with a negative correlation between the grade of adenoid size and nasalance scores preoperatively and a significant positive correlation between them at 1 month postoperatively. However, no significant correlation was detected at 3 months postoperatively.

**Conclusion:**

Transient hypernasality may develop in some patients after adenoidectomy, especially in children with a larger preoperative adenoid size. However, transient hypernasality generally resolves spontaneously within 3 months.

## Introduction

The adenoid is a lymphoid tissue situated in the midline on the roof and posterior wall of the nasopharynx [1]. Most children have adenoid tissue on the pharyngeal wall in the area of velopharyngeal closure. Many children have velo-adenoidal closure rather than velopharyngeal closure. In this type of closure, the separation between the oral cavity and the nasal cavities is produced by the valving effect of the velum toward the pharyngeal wall contacting the adenoid [2]. Adenoidectomy, either alone or with tonsillectomy, is a common procedure in the field of pediatric otorhinolaryngology [3]. Indications for adenoidectomy include nasal obstruction, obstructive sleep apnea, otitis media with effusion, and malformation of craniofacial development. Several techniques can be used to remove adenoids, including curettage, microdebrider, cautery, and coblation. Complications of these procedures include postoperative pain, hemorrhage, edema, and velopharyngeal insufficiency (VPI), and consequently, hypernasality and nasopharyngeal stenosis [4, 5].

Children with adenoid hypertrophy commonly have speech disorders [6], and often present with hyponasality as the adenoid dampens the nasal resonance of speech. Adenoidectomy may result in hypernasality caused by excessive nasal resonance, which is usually transient and due to sudden palatopharyngeal disproportion. However, in rare cases, it can be permanent if there is an anatomical abnormality [5]. Contraction of the soft palate against the adenoid prevents air from escaping into the nasopharynx. Adenoidectomy enlarges the velopharyngeal port, which results in hypernasality [7]. Hypernasality is a type of excessive nasal tone of speech caused by incomplete velopharyngeal closure and is characterized by abnormal sound transmission through the nose during the production of vowels and consonants [8]. The exact incidence of post-adenoidectomy transient hypernasality is unknown; however, permanent hypernasality that may need surgical intervention has been reported to affect between 1:1200 and 1:3000 [9]. Children with normal palatal and velopharyngeal structures are not supposed to develop hypernasality after adenoidectomy. However, tongue and soft palate position, as well as the vocal quality and resonance functions, may be altered postoperatively [10]. As such, some children may have temporary hypernasality after adenoidectomy. The incidence and duration of this alteration are not well known [9, 11]. The speech-related consequences of adenoidectomy have been discussed previously; however, there is a lack of knowledge about the implications of the preoperative adenoid size on postoperative nasal resonance. Therefore, we sought to investigate whether our hypotheses that transient post-adenoidectomy hypernasality is related to the preoperative adenoid size in children with a normal palate and that the condition can settle spontaneously as the palate compensates are correct. If so, preoperative counseling regarding the condition could be provided to patients and their caregiver(s) to alleviate any concerns. The study aimed to compare the preoperative and postoperative nasal resonance of speech in children with adenoid hypertrophy who are candidates for adenoidectomy and identify the relationship between post-adenoidectomy hypernasality and preoperative adenoid size in children with a normal palate.

## Materials and methods

Seventy-four children with adenoid hypertrophy who were candidates for adenoidectomy were included in this prospective observational study. The patients’ ages ranged between four and nine years (mean of 6.1 ± 1.5 years), and 41 were males and 33 females. The indications for adenoidectomy were either sleep-disordered breathing, middle ear effusion, or both. This study was conducted between January 2022 and December 2022. Children who were candidates for tonsillectomy and who presented with craniofacial anomalies, nasal septum deviation/enlarged obstructive turbinates, tonsillar hypertrophy (grades + 3 and + 4), and/or palatal abnormalities were excluded from the study. Parental informed consent was obtained for all patients, and we adhered to the principles outlined in the Declaration of Helsinki. The protocol of the study was approved by the research ethics committee of our institute (N-140-2022).

### Otolaryngologic examination

All patients underwent ear, nose, and throat examinations to exclude craniofacial anomalies. An ear examination was performed to detect middle ear effusion, and an oropharyngeal examination was conducted to assess the palate and tonsillar size and exclude any other cause of airway obstruction. Two patients were excluded from the study as they had tonsillar hypertrophy grade + 3, which may interfere with the results. In addition, one patient was lost to follow-up and was excluded. Therefore, a total of 71 patients were finally included in the study.

### Assessment of adenoid size

All the patients underwent flexible nasopharyngoscopy. We used a flexible nasopharyngoscope, and Tele pack compact endoscopy (Karl Storz, Tuttlingen/Germany). Irritable children were sedated using oral midazolam at a dose of 0.5 mg/kg 15 min before the procedure [12]. Sedation was performed in an office-based well-equipped room with monitoring by an anesthesiologist. Preoperative sedation with midazolam was approved to decrease children’s anxiety, and it may also help to lower the incidence of postoperative nausea and vomiting [13, 14]. The size of the adenoids was graded from 1 to 4, according to the percentage of occupation of the nasopharynx by the adenoid tissue, where grades 1, 2, 3, and 4 refer to the adenoid tissue occupying < 25%, > 25 to  < 50%, > 50 to < 75%, and > 75% of the nasopharyngeal cavity, respectively [15].

### Preoperative assessment of speech

All patients underwent the same assessment protocol at the Phoniatrics unit, including subjective and objective speech assessments using auditory perceptual assessment (APA) and nasometric assessment, respectively. The APAs were performed by an expert phoniatrician (FHM). The evaluation was done using speech samples to determine nasal resonance on a scale of three parameters: normal (0), hyponasality (1), and hypernasality (2) [16]. Nasometric assessment was done by HAFA, using a nasometer (Model-6200; Kay-Elemetrics Corp., Lincoln Park, NJ, USA). The nasalance scores for both oral and nasal sentences were measured, while the child repeated phonetically balanced speech elements with a nasal sentence (/mama betnæjem mænæl/) and oral sentence (/ʕæLi: raħ Jelʕæb ko:ra/) [17]. The examiner did not speak during the examination to avoid derangement, and every child was instructed to repeat the sentences several times before the examination, then asked to speak each one separately (oral and nasal sentences) while wearing a headset device. The nasalance score was calculated using the following equation: nasalance % = nasal energy/(nasal + oral energy)% [18].

### Adenoidectomy

Under general anesthesia with oral endotracheal intubation, the removal of the adenoids was done using the conventional curettage technique. A nasopharyngeal pack was inserted for several minutes, and patients with middle ear effusion were subjected to myringotomy and ventilation tube insertion. After removal of the nasopharyngeal pack, trans-oral endoscopic examination of the nasopharynx with the mouth opened by Boyle-Davis mouth gag was performed, along with retraction of the soft palate by two rubber catheters and a 70ͦ Hopkins 4 mm rigid nasal endoscope was introduced orally. This was done for the detection and removal of any adenoid tissue remnants. Once the patients woke up, the endotracheal tube was removed, and the patients were placed in a lateral position and moved to the recovery unit.

### Postoperative assessment of speech

APA and nasometric assessments were performed 1 and 3 months postoperatively using the same items employed preoperatively and by the same physician.

### Statistical analysis

The data were analyzed using the Statistical Package of Social Science (version 28). The normality of the data was tested using the Kolmogorov–Smirnov single sample test. The quantitative data were presented as the median and range. The Friedman test was used to compare the data between the different time periods and within the treated groups. A post hoc test was used for pairwise comparison, and the data were compared between the groups using the Mann–Whitney test. A *p* value less than 0.05 was considered statistically significant.

## Results

**Fig. 1:**
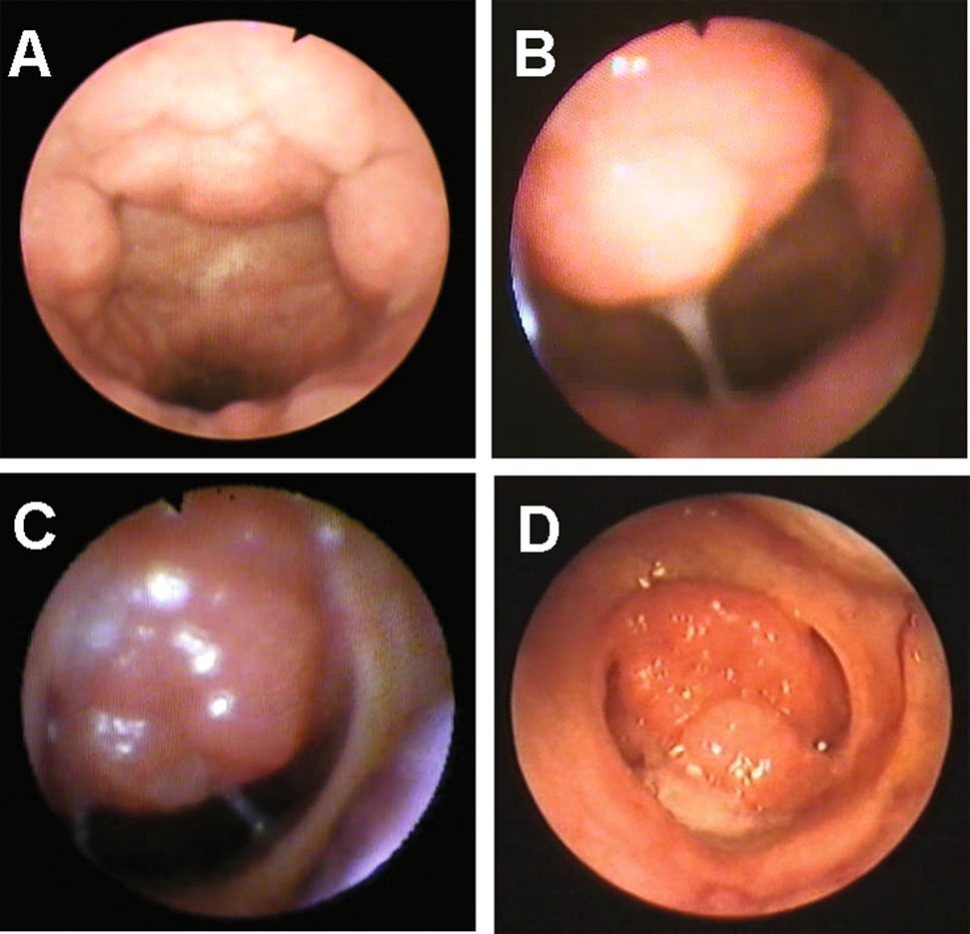
Flexible nasopharyngoscopic examination of the nasopharynx showing the adenoid size. **A** Grade 1 adenoid hypertrophy, **B** grade 2 adenoid hypertrophy, **C** grade 3 adenoid hypertrophy, **D** grade 4 adenoid hypertrophy

Seventy-one children with adenoid hypertrophy were enrolled in the study. Flexible nasopharyngoscopy was used to assess the adenoid size preoperatively. Eleven children needed sedation with oral midazolam. Adenoid hypertrophy was detected as grades 1, 2, 3, and 4 in 15, 21, 23, and 12 patients, respectively (Fig. 1). All patients underwent conventional curettage adenoidectomy, and 28 had myringotomy with ventilation tube insertion. No serious intraoperative or postoperative complications occurred. However, one child with a grade 2 sized adenoid developed postoperative nasal regurgitation of fluids that resolved spontaneously within a week.

**Fig. 2:**
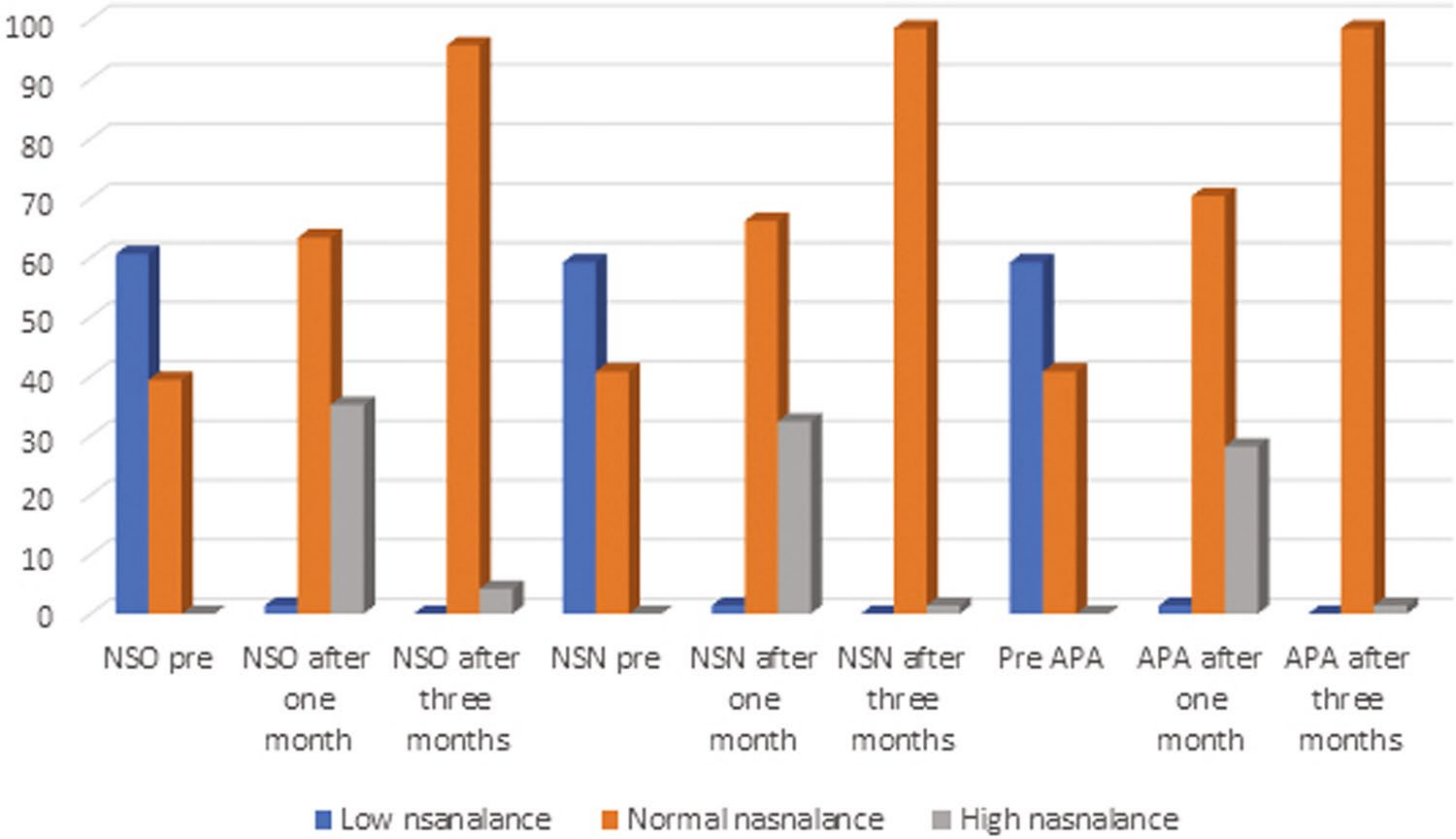
Patients’ speech nasality in percentage as assessed by auditory perceptual assessment and nasometry. *NSO* nasalance score for oral sentences, *NSN* nasalance score for nasal sentences, *pre* preoperative, *APA* auditory perceptual assessment

Preoperative APA showed hyponasality in 42 patients (59.1%), which was significantly related to the adenoid size (*p* < 0.001) (Fig. 2). Patients with adenoid hypertrophy grades 3 and 4 demonstrated more hyponasality (35/33, 94.2%) than patients with grades 1 and 2 (36/9, 25%). One month postoperatively, only one patient showed hyponasal speech because of allergic rhinitis, which improved with steroid nasal spray. In contrast, the speech of 19 patients (26.7%) turned hypernasal. Postoperative hypernasality was found to be significantly related to the preoperative adenoid size (*p* < 0.001). Patients with adenoid hypertrophy grades 3 and 4 showed more hypernasality (35/18, 51.4%) than those with adenoid hypertrophy grades 1 and 2 (36/1, 2.7%). Three months postoperatively, all patients demonstrated normal nasality except for one who was subjected to speech therapy and a longer follow-up period due to persistent hypernasality (71/1, 1.4%).

Nasalance scores for both oral (NSO) and nasal (NSN) sentences (Fig. 2) showed significant differences among the three time periods. According to the pairwise comparison, significant differences were found between the scores of NSO and NSN preoperatively and 1 month postoperatively (*p* < 0.001 for both) and between the scores of NSO and NSN preoperatively and 3 months postoperatively (*p* < 0.001 for both). In addition, there was a significant difference between the scores of both items at 1 and 3 months postoperatively (*p* = 0.001 for both). A statistically significant negative correlation was found between the grade of the adenoid size and the scores of NSO and NSN preoperatively (*r* = − 0.850, *p* < 0.001; *r* = − 0.833, *p* < 0.001, respectively). A statistically significant correlation was found between the grade of the adenoid size and the scores of both items (NSO and NSN) at 1 month postoperatively (*r* = 0.392, *p* = 0.001; *r* = 0.434, *p* < 0.001, respectively). However, there was no significant correlation between the adenoid size and the NSO and NSN at 3 months postoperatively (*r* = 0.131, *p* = 0.275; *r* = 0.120, *p* = 0.320, respectively) (Table 1).

**Table 1:** Correlation between nasalance scores and adenoid size

	Adenoid size	
	r	*p* value
NSO preoperative	− 0.850	< 0.001*
NSO postoperative 1 month	0.392	0.001*
NSO postoperative 3 months	0.131	0.275
NSN preoperative	− 0.833	< 0.001*
NSN postoperative 1 month	0.434	< 0.001*
NSN postoperative 3 months	0.120	0.320

*NSO* nasalance score for oral sentences, *NSN* nasalance score for nasal sentences

*r* correlation coefficient

*Significant *p* value < 0.05

## Discussion

Hypertrophy of the adenoid tissue often occurs at a young age and can cause narrowing of the nasopharyngeal airway space, harbor organisms and become a reservoir for pathogens that can cause rhinosinusitis and otitis media with effusion [19]. Removal of this hypertrophied tissue is a common surgical operation that is usually performed on an outpatient basis by an otolaryngologist. Adenoidectomy is considered a simple straightforward, and relatively short procedure, so any recognized complications may have significant implications [20]. Indeed, hypertrophied adenoids may decrease the anteroposterior distance between the soft palate and the posterior pharyngeal wall, which makes velopharyngeal closure easier. Palato-adenoidal contact during speech occurs in the presence of an enlarged adenoid instead of the normal palatopharyngeal contact. Removal of this hypertrophied adenoid tissue may lead to sudden widening of the velopharyngeal port, which requires further posterosuperior displacement of the soft palate to compensate. This condition leads to the escape of air into the nose during speech articulation with more resonance, especially on the production of plosive consonant phonemes, a problem called ‘hypernasality’ [5, 20].

Post-adenoidectomy hypernasality may be transient or permanent. The transient type has been attributed to postoperative edema and pain, which causes spasms of the palatal muscles [21]. However, some authors have reported that transient hypernasality may be present for several months [5, 22, 23], so factors other than postoperative edema and pain could be involved in the occurrence of this postoperative problem. We first conceived this study after noticing transient speech hypernasality in many children after the removal of their adenoids. The current study included 71 children who underwent adenoidectomy. We assessed their speech nasality before and after the operation subjectively by APA and objectively by nasometry. We detected preoperative hyponasality in 59.1% of patients by APA and in 60.6 and 59.2% by nasometry for NSO and NSN, respectively. These results were expected, as many authors have reported that adenoid hypertrophy is a leading cause of hyponasal speech [4, 6, 24]. Postoperatively, hyponasality was relieved in all patients except for one with allergic rhinitis, which improved with treatment.

One month postoperatively, we identified hypernasality in 26.7% by APA and 35.2 and 32.4% for NSO and NSN by nasometry, respectively. However, the nasality of all patients had improved by the third month except for in one who needed a longer follow-up period. Parton and Jones [20] reported that transient hypernasality is a relatively common event following adenoidectomy, and early follow-up may result in an overestimation of its incidence. However, Khami et al. [9] reported that the incidence of transient post-adenoidectomy hypernasality is not well known, as patients with transient problems are unlikely to return for treatment. Previous studies have included patients who sought treatment for hypernasality after adenoidectomy, so their speech did not resolve spontaneously, and thus transient hypernasality may be underestimated, and the actual incidence cannot be known without prospective studies. Mushi et al. [25] found that adenoidectomy is a cause of 10% of VPI in non-cleft patients, showing that the procedure is not a rare cause of hypernasality.

A patient with submucous cleft palate, short palate, abnormal deep nasopharynx, or neuromuscular disorder involving the velopharyngeal port may develop post-adenoidectomy VPI with persistent hypernasality and even nasal regurgitation of food and fluid that could need surgical intervention [19, 21]. In such patients, the adenoids may act as a mattress against the soft palate, assisting velopharyngeal closure and compensating for poor palatal mobility. Following adenoidectomy, compensation is eliminated, and VPI may develop [26]. Therefore, such patients should not undergo adenoidectomy unless absolutely indicated, and in these situations, conservative or partial adenoidectomy should be performed [27, 28].

Many studies have investigated the effect of adenoidectomy on postoperative hypernasality [5–7]. The current study investigated the effect of adenoid size on post-adenoidectomy hypernasality. We found a significant relationship between the grade of adenoid hypertrophy and the occurrence of postoperative hypernasality, as patients with adenoid hypertrophy grades 3 and 4 showed more hypernasality than those with adenoid size grades 1 and 2. Although the problem is usually transient, it may cause parental annoyance postoperatively.

The findings of the current study support the hypothesis that there is a relationship between adenoid size and the development of post-adenoidectomy transient hypernasality. Therefore, otolaryngologists should provide preoperative counseling to the parents/caregivers about the possibility of their child developing this transient problem, especially in children with large adenoid sizes. This kind of hypernasality in children with a normal palate might last for a few days to months and then resolve spontaneously.

The current study has highlighted several areas that require further investigation. Further prospective studies with a larger sample size should be conducted to detect the incidence of transient hypernasality after adenoid removal. In addition, the effect of providing conservative techniques to facilitate rapid improvement of the condition and various adenoidectomy techniques on the development of transient hypernasality in children with larger grades of adenoid size should be investigated.

There are several limitations to the current study. First, each finding was assessed by a single observer, so we could not assess the inter-rater reliability, limiting the power of the study. Also, children with allergic rhinitis, which is a common pediatric illness and can cause nasal obstruction and hyponasal speech, were not excluded from the study, which could have introduced bias.

## Conclusion

Although adenoid hypertrophy is a cause of hyponasal speech, adenoidectomy may result in hypernasality that is usually transient in patients with a normal palate. The larger the adenoid size, the more likely it is for hypernasality to occur after adenoidectomy. Preoperative counseling should be provided to inform the family that their child may develop temporary hypernasality after adenoidectomy, particularly in cases with a large grade of adenoid size, with the assurance that the condition is likely to resolve spontaneously without intervention within a few months.

## Data Availability

The data that support the findings of this study are available on request from the corresponding author (AM). The data are not publicly available due to their containing information that could compromise the privacy of research participants.
